# Assessment of HBV flare in a randomized clinical trial in HIV/HBV coinfected subjects initiating HBV-active antiretroviral therapy in Thailand

**DOI:** 10.1186/1742-6405-9-6

**Published:** 2012-03-09

**Authors:** Anchalee Avihingsanon, Gail V Matthews, Sharon R Lewin, Pip Marks, Jose Sasadeusz, David A Cooper, Scott Bowden, Stephen Locarnini, Greg J Dore, Kiat Ruxrungtham

**Affiliations:** 1 HIV-NAT Thai Red Cross AIDS Research Centre, Bangkok, Thailand; 2 Division of Allergy and Clinical Immunology, Faculty of Medicine, Bangkok, Thailand; 3 The Kirby Institute for infection and immunity in society, University of New South Wales, Sydney, Australia; 4 Infectious Diseases Unit, The Alfred Hospital, Melbourne, Australia; 5 Department of Medicine, Monash University, Melbourne, Australia; 6 Centre for Virology, Burnet Institute, Melbourne, Australia; 7 Victorian Infectious Diseases Service, Royal Melbourne Hospital, Melbourne, Australia; 8 Victorian Infectious Diseases Reference Laboratory, Melbourne, Australia; 9 The Kirby Institute for infection and immunity in society, University of NSW, 376 Victoria Street, Darlinghurst, Sydney, NSW 2010, Australia

**Keywords:** Hepatitis B, HIV, Antiretroviral therapy, Asia, Hepatic flare, Hepatotoxicity

## Abstract

**Abstract:**

**Background**

Hepatic Flare (HF) after initiation of highly active antiretroviral therapy (HAART) in HIV-HBV coinfected individuals is well recognized but prospective data on predictors and subsequent outcome are limited.

**Methods**

The Tenofovir in HIV-HBV coinfection study was a randomized clinical trial of HBV-active HAART including lamivudine and/or tenofovir in antiretroviral naïve HIV-HBV individuals in Thailand.

**Results**

Early HF (EHF) was defined as ALT > 5 × ULN during the first 12 weeks. EHF was observed in 8 (22%) of individuals at a median of 56 days. 6/8 EHF cases were asymptomatic and resolved with HAART continuation, however one subject with underlying cirrhosis died following rapid hepatic decompensation. EHF was significantly associated with higher baseline ALT (79 IU/L vs 36 IU/L non-EHF, *p *= 0.008) and HBV DNA (9.9 log_10 _c/ml vs 8.4 log_10 _c/ml non EHF, *p *= 0.009), and subsequent serological change. HBeAg loss occurred in 75% of EHF cases versus 22% in non-EHF (*p *= 0.04), and HBsAg loss in 25% of EHF cases versus 4% of non-EHF (*p *= 0.053).

**Conclusion**

EHF after HBV active HAART initiation was frequently observed in this population. Timing of EHF, association with elevated ALT and HBV DNA and high rate of seroconversion are all consistent with immune restoration as the likely underlying process.

**Clinical Trial number:**

NCT00192595.

## Background

Hepatotoxicity (HT) after HAART initiation has been reported in 2-14% of HIV positive individuals [1-3], and the risk increases significantly in HBV or HCV coinfected individuals [1,4]. Definitions of HT vary but the most commonly used is based on the AIDS Clinical Trial Group (ACTG) criteria in which an increase in ALT and/or AST above 5 × upper limit of normal (ULN) (Grade 3) is defined as severe HT. HF (HF) appears to be particularly common within the first months after HAART initiation, suggesting a potential immunological component to its development. The outcome following HF may be beneficial with subsequent serological change; on the other it may also be associated with morbidity, and even mortality. Although, HF after initiation of HAART in HIV-HBV coinfected individuals is well recognized, prospective data on predictors and subsequent outcome are limited.

We therefore examined the incidence, characteristics, predictors and subsequent outcome of HF occurring in the first 12 weeks after HAART initiation (early HF [EHF ]) within the Tenofovir in Coinfection study (TICO), a randomized clinical trial of HBV-active HAART including lamivudine and/or tenofovir in 36 antiretroviral (ARV) naïve HIV-HBV individuals in Thailand.

## Methods

Full eligibility criteria for this study are given elsewhere [5], but in brief, subjects were HIV-1 antibody positive and naïve to ARV with chronic hepatitis B infection (HBV surface antigen (HBsAg)-positive > 6 months apart with HBV DNA > 100,000 copies/mL). Subjects were excluded if they had evidence of other causes for chronic liver disease ie hepatitis C virus, acute hepatitis A virus, autoimmune disease, serum ALT level > 1000 IU/L. Early HF was defined as ALT > 5 × ULN during the first 12 weeks.

Liver function tests (LFTs) were routinely performed at weeks 0, 4, 8, 12, 24, 36 and 48. Plasma for HBV DNA testing and peripheral blood mononuclear cells (PBMC) were collected at weeks 0, 4, 8 and 12, 24 and 48 and stored at -80 C and -140 C respectively. HBV DNA levels were analysed at the Victorian Infectious Diseases Laboratory (VIDRL), Melbourne, Australia. HBV DNA measurement was performed by the Versant HBV DNA 3.0 bDNA assay (Siemens HealthCare, Tarrytown, NY, USA). The linear dynamic range of the assay was from 2 × 10^3 ^- 1 × 10^8 ^copies/mL.

All subjects gave written informed consent and the relevant Human Research Ethics Committees in Thailand and Australia approved the study.

### Statistical analysis

Cases were identified using the pre-defined criteria as stated above and compared with non-cases in regard to baseline characteristics using standard descriptive statistics. Differences between HF and non-HF cases were compared using the Mann-Whitney U-test or Chi-square test. Univariate analyses were performed to identify variables associated with development of hepatic flare. Data were analysed using STATA (StataCorp, College Station, Tx, USA).

## Results

Thirty-six advanced HIV/HBV co-infected patients with a median CD4 count of 36 cells/mm^3 ^(interquartile range (IQR) 19-208 cells/mm^3^) and HIV RNA of 4.7 log_10 _c/ml (IQR 4.3-5.1 log_10 _c/ml) were enrolled. HBV DNA was high at baseline (median 8.4 log_10 _c/ml, IQR 8.1-9.5 log_10 _c/ml), 61% of the group were HBeAg positive and 53% had normal baseline ALT. Twenty-nine patients (81%) were infected with HBV genotype C. Seven (19%) had experienced a prior AIDS defining illness, six (17%) were on isoniazid and rifampicin for tuberculous treatment. Twenty-four (67%) and 58% were on co-trimoxazole and fluconazole, respectively, for opportunistic infection (OI) prophylaxis.

EHF was observed in 8 (22%) individuals at a median of 56 days (range 21-86 days) with a median peak ALT of 395 IU/L (range 178-2560 IU/L). Six of the 8 EHF cases were asymptomatic and resolved with continuation of HAART. In this group, 3/4 HBeAg positive subjects subsequent lost HBeAg; one also experienced HBsAg loss and another had HBsAg loss with anti-HBs seroconversion, all had ALT < 2 × ULN and undetectable HBV DNA (< 200 c/ml) at week48.

Two cases of EHF were clinically symptomatic, one of whom had underlying cirrhosis and died following rapid hepatic decompensation. The other was a 37 yr old HBeAg negative man with a baseline CD4 count of 20cells/mm^3^, HBV DNA of 10.0 log_10_c/ml and baseline ALT of 78 IU/L. He commenced a regimen of AZT/3TC/EFV. At week 8 the subject presented with nausea and jaundice and was found to have an ALT of 2560 IU/L. Efavirenz was switched to lopinavir/ritonavir and HAART was continued with close observation. LFTs subsequently improved and by week 48 had normalised with undetectable HIV and HBV viral load but no loss of HBsAg. After week48, EFV was subsequently reintroduced with no further rise in transaminase values.

Baseline characteristics of EHF cases and non-EHF cases are given in Table 1.

**Table 1:** Univariate analysis of predictors of early hepatic flare.

	EHF (n = 8)	Non-EHF (n = 28)	*p*-value
Age yrs (median, IQR)	37 (33-42)	33 (28-40)	0.25

Male gender	88%	43%	0.12

HBeAg positive	50%	64%	0.47

Alcohol (> 3units/day)	25%	25%	1.00

Cirrhosis	20%	42%	0.36

HBV Genotype C	75%	82%	0.28

BMI kg/m^2 ^(median. IQR)	19 (18-20)	20 (18-22)	0.20

BL CD4 cells/mm^3 ^(median. IQR)	52 (18-131)	32 (19-214)	0.96

BL HIV RNA log_10 _c/ml (median. IQR)	4.9 (4.7-5.1)	4.7 (4.3-5.1)	0.22

BL ALT IU/L (median. IQR)	79 (59-96)	36 (22-59)	0.008*

BL HBV DNA log_10 _c/ml (median. IQR)	9.9 (8.4-10.4)	8.4 (7.8-9.0)	0.009*

Randomised HBV therapy	3TC n = 5	3TC n = 8	0.193
	TDF n = 2	TDF n = 10	
	3TC/TDF n = 1	3TC/TDF n = 10	

OI prophylaxis (fluconazole and/or cotrimoxazole)	88%	61%	0.162

Izoniazid therapy	0%	21%	0.302

All comparisons made using the Mann-Whitney U-test except categorical variables where Chi-squared or Fischers Exact test used

*Statistically significant at *p *< 0.05

EHF cases had significantly higher baseline ALT (79 IU/L vs 36 IU/L, *p *= 0.008) and HBV DNA (9.9 log_10 _c/ml vs 8.4 log_10 _c/ml, *p *= 0.009) than non-EHF cases.

There were no differences between the two groups for age, gender, HBV genotype, HIV related parameters (CD4 count and HIV RNA) or use of potentially hepatotoxic agents (izoniazid/rifampicin/fluconazole/cotrimoxazole/alcohol) prior to HAART initiation. Similarly no significant differences were observed by randomised therapy, although EHF was seen in 38% of subjects in the arm containing 3TC alone versus 15% of subjects on TDF-containing regimens (*p *= 0.078).

**Figure 1:**
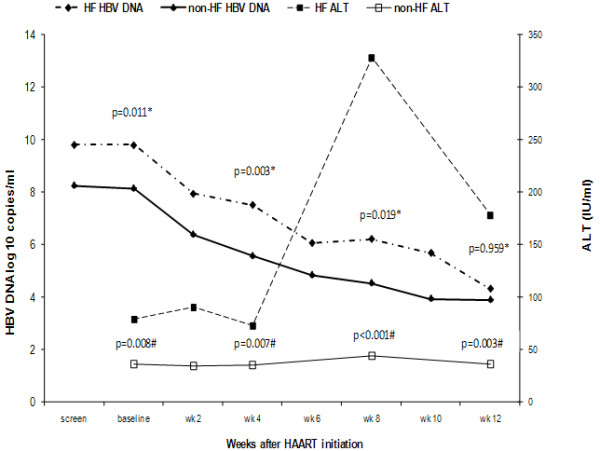
**ALT and HBV DNA values in HF and non-HF subjects over 1^st ^12 weeks after HAART initiation**. -axis: Weeks after HAART initiation. y-axis 1: HBV DNA log _10 _c/ml. y-axis 2: ALT IU/L.

ALT and HBV DNA remained significantly higher in the EHF group throughout the first 12 weeks of therapy (Figure 1) although by week 12 the difference in HBV DNA between groups had become non-significant (4.31log_10_c/ml vs 3.88 log_10 _c/ml, *p *= 0.96). The reduction in HBV DNA between weeks 0 and 12 was significantly greater in the subjects who experienced HF than those that did not (*p *= 0.039). ALT remained significantly higher in the EHF group at week 12 of therapy (median 153 IU/L vs 36 IU/L, *p *= 0.03) but was similar to the non-EHF group by wk 24 (median 31 IU/L vs 43 IU/L, *p *= 0.26) and all time points thereafter.

EHF was associated with subsequent HBV serological change. HBeAg loss occurred in 75% of EHF cases versus 22% in non-EHF (*p *= 0.04), and HBsAg loss in 25% of EHF cases versus 4% of non-EHF (*p *= 0.053). In fact, the case of HBsAg loss in the non-EHF group was a subject who experienced a late HF with an ALT rise to 254 IU/L at week 24 having previously been normal. Follow-up serological testing demonstrated anti-HBe seroconversion and HBsAg loss in this subject. This was the only other subject in the study to experience HF after HAART initiation, giving an overall rate of HF of 25% over 48 weeks. Both HBeAg and HBsAg loss were significantly predicted by the occurrence of any HF. Among HBeAg positive subjects, 80% of cases with HF at any time point experienced HBeAg loss compared with 18% in those without HF (*p *= 0.009), and among all subjects 33% of HF cases experienced HBsAg loss compared to 0% in subjects without any HF (*p *= 0.002).

## Discussion

EHF after HBV active HAART initiation was frequently observed in this population.

We found that elevated ALT and higher HBV DNA at baseline are significant predictors of EHF. In addition the occurrence of EHF was positively associated with subsequent HBeAg and HBsAg loss, both of which occurred at elevated rates (33% and 8% respectively) during the 48-week study follow-up.

Elevated transaminases in the setting of HAART and viral hepatitis co-infection are commonly multifactorial and may reflect natural alterations in hepatitis disease activity, direct antitretroviral hepatotoxicty, drug hypersensitivity, co-existent hepatotoxic medication, alcohol excess and immune restoration disease. The reasons for the high rate of flare observed in our prospective clinical trial are unclear but can be hypothesized.

Our population had advanced immunodeficiency with a median pre-HAART CD4 count of only 36 cells/mm^3 ^and high usage of potentially hepatotoxic medications including cotrimoxazole, fluconazole and isoniazid/rifampicin - however none of these factors were identified as associated with EHF in univariate analysis. Similarly, no difference was observed in baseline level of alcohol use between groups (Table 1). Nucleoside analogue agents are also potentially hepatotoxic, but usually through the development of mitochondrial dysfunction and microvesicular hepatic steatosis - this was felt to be unlikely in our subjects given the time frame in which the HF events were observed and the absence of associated features such as lactic acidosis suggesting mitochondrial dysfunction. All subjects were prescribed EFV and EFV associated hepatotoxicity has been clearly described, although at a lesser rate than with nevirapine [4,6]. EFV induced hepatotoxicity as a cause of HF is possible in the symptomatic case, (although subsequent EFV reintroduction without HF at a later date makes this unlikely); and the possibility of EFV-induced hepatotoxicity as a cause of death in the case with cirrhosis is also conceivable.

Overall however, timing of EHF, association with elevated ALT and HBV DNA and high rate of seroconversion are all consistent with immune restoration stimulating HBV-specific immune responses as the likely underlying process

Spontaneous flares in CHB are well recognised and reflect the dynamic nature of the balance between immunological control and HBV replication [7]. Sometimes, but not universally, these flares will be followed by HBeAg loss, anti-HBe seroconversion and even occasionally HBsAg loss/seroconversion. In the context of HBV treatment and effective HBV DNA reduction the rate of HF increases, particularly in the early months of treatment [8,9]. These flares likely reflect an underlying shift in host-viral balance with some regeneration of host T-cell responsiveness as HBV DNA decreases [10], but are still accompanied by anti-HBe seroconversion in around only 20% of subjects by 1 year [8].

In the setting of HIV coinfection the situation is enhanced by the simultaneous reduction in HIV RNA observed with effective HAART and the subsequent restoration of either an adaptive or innate response to HBV antigens. The concept of immune restoration disease (IRD) is characterised by the finding of rapid restoration in immunity in the presence of a high pathogen load resulting in abnormal and exaggerated immune responses, often mediated by high levels of interferon gamma [11-13]. Besides elevated ALT, our study identified higher HBV viral load both at baseline and throughout the early weeks of therapy, as a significant predictor of EHF, a finding consistent with the theory of IRD and high antigen burden as a cause for flare in these subjects. This theory is supported by our findings of elevated CXCL10, an interferon stimulated gene, in patients with EHF within this study [14].

The most striking aspect of our study however is the link between HF and the high rate of subsequent seroconversion. In a retrospective study examining the role of 3TC-containing HAART on serological change in 82 HIV-HBV coinfected subjects, Miailhes et al reported an anti-HBe seroconversion rate of 17% and an overall HBsAg loss rate of 6% during a median follow-up of 5 years [15]. Interesting, they found more advanced HIV CDC stage at baseline to be associated with subsequent seroconversion rates. HBsAg and/or HBeAg seroconversion occurred in 29% of stage C subjects versus only 3% of patients with stage A disease, again suggesting a major role for immunorestoration in promoting seroconversion. One specific difference in our study to the majority of reports on outcomes in HIV-HBV coinfected individuals commencing HAART is that our population was Asian with predominantly Genotype C and likely vertically acquired HBV disease. Thus differences in the virus, host and/or natural history of HBV in this population may all be contributing to our findings. Given the overwhelming greater burden of HBV disease in Asian countries, further study of the mechanisms and outcomes of HBV-active HAART in this population are certainly required.

Our study is undoubtedly limited by the relatively small number of subjects to elucidate further any additional associations or conclusions. However, it is one of the few randomised clinical trials performed in this population.

## Conclusion

The rate of HF is high during the first 12 weeks of HBV-active HAART initiation in an advanced immunodeficient Asian population. Although the mechanisms underlying this may be multifactorial, our findings most strongly support a significant role for HBV immune restoration disease as the major contributing factor. A strategy of short-course lead-in anti-HBV therapy (using drugs without anti-HIV activity) could be studied as a potential mechanism to decrease HBV burden and prevent HF after HAART initiation in such individuals.

## Competing interests

The Kirby Institute for infection and immunity in society is funded by the Australian Government Department of Health & Ageing and is affiliated with the Faculty of Medicine, The University of New South Wales. KR and Vaccine & Cellular Immunology Laboratory are partly funded by the Research-team Strengthening Grant, National Center for Genetic Engineering and Biotechnology (BIOTEC), Thailand. Other authors declare that they have no competing interests.

## Authors' contributions

All authors participated in the design of the study. AA and GVM drafted the manuscript. JS, SC and SL performed the laboratory tests. SRL, PM, DAC, GJD and KR reviewed the paper. All authors have seen and approved the final manuscript.
